# Comparative Genomics among Closely Related *Streptomyces* Strains Revealed Specialized Metabolite Biosynthetic Gene Cluster Diversity

**DOI:** 10.3390/antibiotics7040086

**Published:** 2018-10-02

**Authors:** Cláudia M. Vicente, Annabelle Thibessard, Jean-Noël Lorenzi, Mabrouka Benhadj, Laurence Hôtel, Djamila Gacemi-Kirane, Olivier Lespinet, Pierre Leblond, Bertrand Aigle

**Affiliations:** 1 Université de Lorraine, INRA, DynAMic, F-54000 Nancy, France; claudia.morgado-vicente@univ-lorraine.fr (C.M.V.); annabelle.thibessard@univ-lorraine.fr (A.T.); mabrouka.benhadj@gmail.com (M.B.); laurence.hotel@univ-lorraine.fr (L.H.); 2 Institute for Integrative Biology of the Cell (I2BC), CEA, CNRS, University Paris-Sud, University Paris-Saclay, Gif-sur-Yvette CEDEX, France; jean-noel.lorenzi@i2bc.paris-saclay.fr (J.-N.L.); olivier.lespinet@i2bc.paris-saclay.fr (O.L.); 3 Biomolecules and Application Laboratory, Faculty of Exact Sciences and Natural and Life Sciences, University of Tebessa, Tebessa 12002, Algeria; 4 Department of Biochemistry, Faculty of Science, University Badji Mokhtar Annaba, Annaba 23000, Algeria; dj_gacemi@yahoo.fr

**Keywords:** *Streptomyces*, strain, specialized metabolites, biosynthetic gene cluster

## Abstract

Specialized metabolites are of great interest due to their possible industrial and clinical applications. The increasing number of antimicrobial resistant infectious agents is a major health threat and therefore, the discovery of chemical diversity and new antimicrobials is crucial. Extensive genomic data from *Streptomyces* spp. confirm their production potential and great importance. Genome sequencing of the same species strains indicates that specialized metabolite biosynthetic gene cluster (SMBGC) diversity is not exhausted, and instead, a pool of novel specialized metabolites still exists. Here, we analyze the genome sequence data from six phylogenetically close *Streptomyces* strains. The results reveal that the closer strains are phylogenetically, the number of shared gene clusters is higher. Eight specialized metabolites comprise the core metabolome, although some strains have only six core gene clusters. The number of conserved gene clusters common between the isolated strains and their closest phylogenetic counterparts varies from nine to 23 SMBGCs. However, the analysis of these phylogenetic relationships is not affected by the acquisition of gene clusters, probably by horizontal gene transfer events, as each strain also harbors strain-specific SMBGCs. Between one and 15 strain-specific gene clusters were identified, of which up to six gene clusters in a single strain are unknown and have no identifiable orthologs in other species, attesting to the existing SMBGC novelty at the strain level.

## 1. Introduction

Microorganisms are unmatched in their capacity to produce chemically diverse specialized metabolites [1]. *Streptomyces* in particular, which are ubiquitous filamentous bacteria with a differentiating mycelial life cycle, have a significant role in biotechnology industry. They are employed in the production of more than half of all antibiotics used in human and veterinary medicine, as well as a large assortment of other high-value molecules such as immune suppressants, anticancer, and anti-parasitic molecules [2,3]. The increasing incidence of antibiotic resistant infections and the lack of new antimicrobial agents have driven the race for natural product discovery. The significant advances made in high-throughput DNA sequencing, along with the relatively low cost of whole genome sequencing, have greatly contributed to this effort [4]. Strategies to address the antimicrobial shortage include both the intensive screening of uncultivable microorganisms through metagenomics and environmental DNA cloning for heterologous expression [5,6], as well as further genome mining of cultivable isolates. Consequently, correlation data between species and specialized metabolomes are accumulating progressively.

*Streptomyces* spp. are amongst the best-characterized bacteria, and the vastly increasing genomic data that is available has revealed the presence of thousands of specialized metabolite biosynthetic gene clusters (SMBGCs), highlighting their enormous potential for natural product biosynthesis [7,8]. Estimates say that no more than 10% have been identified to date, and furthermore, the majority of these SMBGCs appear to be “silent” [9]. A recent bioinformatic analysis of six *Streptomyces albus* strains led to the identification of strain-specific SMBGCs, speaking to the chemical diversity at the strain level [10]. It has also been shown that *Streptomyces* species with identical 16S rRNA sequences can have distinct specialized metabolomes [11]. Moreover, studies in actinobacteria *Salinispora* demonstrated an important biosynthetic pathway diversity in marine populations, providing insight into new chemical diversity-generating mechanisms [12].

A *Streptomyces* strain collection that included isolates with interesting biological activities against both Gram-positive and Gram-negative indicator strains was recently characterized [13]. Here, we report the genomic analysis of a small group of these environmental *Streptomyces* strains, including those antimicrobial-producing isolates and the associated SMBGC diversity that was encountered. This analysis highlights that the use of closely related strains to identity new SMBGCs in *Streptomyces* constitutes a promising approach for the identification of novel specialized biosynthetic pathways.

## 2. Results and Discussion

### 2.1. Analyzed Strains are Phylogenetically Diverse

The genomes of five environmental strains from a previously constructed *Streptomyces* collection [13], isolates E5N91, E2N166, E2N171, E1N211, E5N298, were sequenced and examined. Draft genome sequences have 7.0–8.5 Mb with an average G+C content of 71.8%, which is in good accordance with the genus (Table 1).

**Figure 1:**
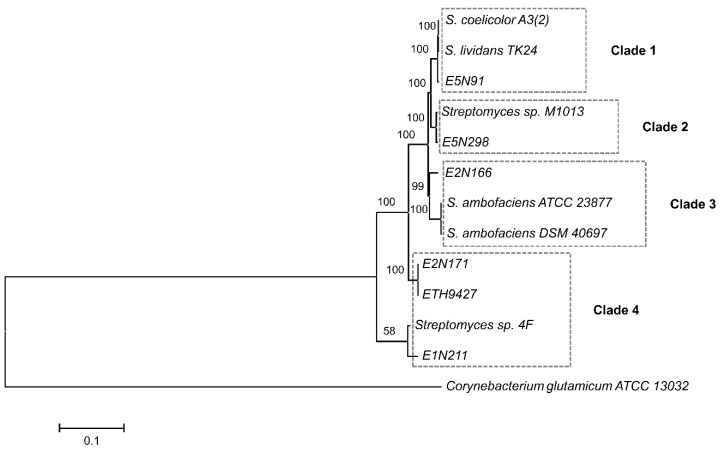
Phylogenetic tree of sequenced isolated and reference strains. Constructed based on the concatenated sequence alignment of five loci (*atpB*-*gyrB*-*recA*-*rpoB*-*trpB*), using inference by neighbor-joining with 100 bootstrap replicates. Only positions with a minimum 92% site coverage were used and the final dataset has 9141 positions in total. The scale bar indicates 10% estimated sequence divergence. The tree is rooted on *Corynebacterium glutamicum* ATCC 13032.

A comparative genomics analysis of the nucleotide sequences, including the taxonomically close *Streptomyces* sp. ETH9427 recently sequenced [14], was carried out through a multilocus sequence typing (MLST) using five genes: *atpD* (ATP synthase, β subunit), *gyrB* (DNA gyrase, subunit B), *recA* (recombination protein), *rpoB* (RNA polymerase, β subunit), and *trpB* (tryptophan synthase, β subunit). It highlighted the taxonomic relationships between the strains and with other model *Streptomyces* species (Figure 1). Four clades are distinguishable in the constructed phylogenetic tree: the first clade with strain E5N91, *Streptomyces coelicolor* A3(2) [7], and *S. lividans* TK24 [15], the second containing strain E5N298 and the recently described *Streptomyces* sp. M1013 [16], clade 3, including strain E2N166 and *S. ambofaciens* strains ATCC 23877 [17] and DSM 40697 [18], and finally strains E2N171, E1N211, and ETH9427 grouped with *Streptomyces* sp. 4F (accession number CP013142.1) in clade 4 (Figure 1).

Taxonomic affiliations were further assessed by calculating the average nucleotide identity (ANI) using JSpeciesWS [19], and are resumed in Table 2. The ANI in clade 1 between strain E5N91 and *S. coelicolor* and with *S. lividans* is 97.32% (with 75.5% of the genome nucleotides aligned) and 97.65% (77.0% of nucleotides aligned), respectively. In clade 2, the ANI value is 98.19% (80.9% nucleotides aligned) between strain E5N298 and *Streptomyces* sp. M1013, and in clade 3 between strain E2N166, *S. ambofaciens* ATCC 23877, and *S. ambofaciens* DSM 40697, the ANI values are 88.86% (65.3% alignment) and 88.75% (with 66.0% nucleotides aligned), respectively. The values in clade 4 are as follows: the E2N171 strain’s ANI value is 98.98% with strain E1N211 (83.5% of aligned nucleotides), 99.14% (84.8% alignment) with strain ETH9427, and 94.56% (76.3% of nucleotides aligned) with *Streptomyces* sp. 4F; the ANI value of strain E1N211 is 99.90% (86.4% of aligned nucleotides) with strain ETH9427, and 94.41% (71.1% alignment) with *Streptomyces* sp. 4F; and the ETH9427 strain’s ANI value is 94.13% (70.7% of nucleotides aligned) with *Streptomyces* sp. 4F. These results suggest that strain E5N91 belongs to a species that is taxonomically closely related to *S. coelicolor* and *S. lividans* in clade 1, as do strain E5N298 and *Streptomyces* sp. M1013 in clade 2. Moreover, the ANI value among the six studied strains varies between 81.40–99.90%, except in those from clade 4, where the ANI value is 99.00–99.90%, well above the species boundary [20] and indicating that strains E2N171, E1N211, and ETH9427 belong to the same species. The closest relative to these strains is *Streptomyces* sp. 4F, and as such, all are clustered in clade 4. However, these strains and *Streptomyces* sp. 4F belong to distinct but related species, as seen by the ANI values between 94.13–94.56% and a low branch bootstrap in the phylogenetic tree (Figure 1). Still, these strains have highly similar 16S ribosomal DNA sequences (dissimilarities between 0.1–1.1%), which would mistakenly suggest that most of the strains belong to the same species. These results indicate that 16S single-gene analysis is not the most accurate tool for streptomycetes phylogeny analysis and inferring intraspecific genetic diversity, as already seen previously [21,22]. Instead, taxonomic relationships are better resolved using genome comparison indexes, such as ANI, and multi-gene analysis, such as MLST [23,24], which in some cases can even match whole-genome comparisons [25].

### 2.2. Specialized Metabolism Diversity

**Figure 2:**
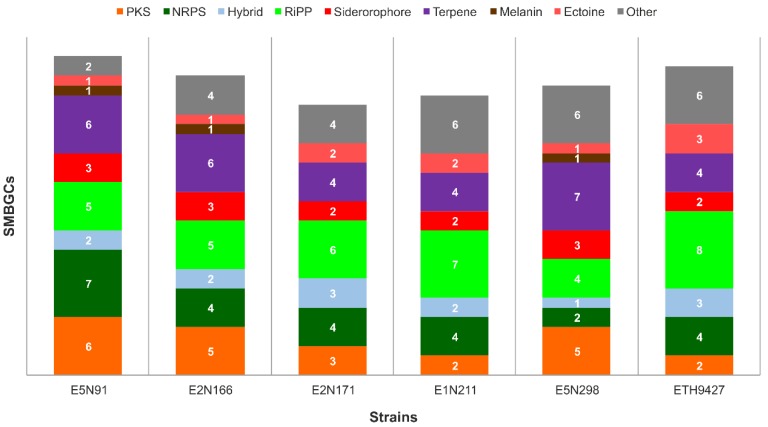
Specialized metabolite biosynthetic gene clusters identified in analyzed strains. Genome sequences were analyzed with antiSMASH 3.0 for gene cluster identification. The “Hybrid” category refers only to PKS-NRPS hybrid gene clusters. Other hybrid clusters that were detected were separated into their constituent parts to better visualize cluster diversity. Clusters encoding bacteriocins, lantipeptides, and lassopeptides are grouped in the category “RiPP”, and the category “Other” includes other KS pathways and less common clusters such as phenazines and butyrolactones, among others.

The identification of SMBGCs was performed using antiSMASH 3.0 [26] and then edited to take into account previously published experimental data. A total of 28–33 SMBGCs was predicted in the isolated strains (Figure 2), with strain E2N171 harboring the least (28) and strain E5N91 harboring the most (33) gene clusters, expectantly coinciding with the smallest and largest genome, respectively. All six strains harbor SMBGCs belonging to the principal classes such as polyketide synthases (PKS), non-ribosomal synthetases (NRPS), and PKS–NRPS hybrids. Interestingly, the analysis revealed that a single strain within its clade can contain up to 48% strain-specific SMBGCs, suggesting that strain-level search for new SMBGCs diversity is not exhausted, and genome sequencing of several strains from a single species can lead to the identification of new gene clusters, as already seen previously [10,12].

### 2.3. The Core and Conserved Specialized Metabolites

**Figure 3:**
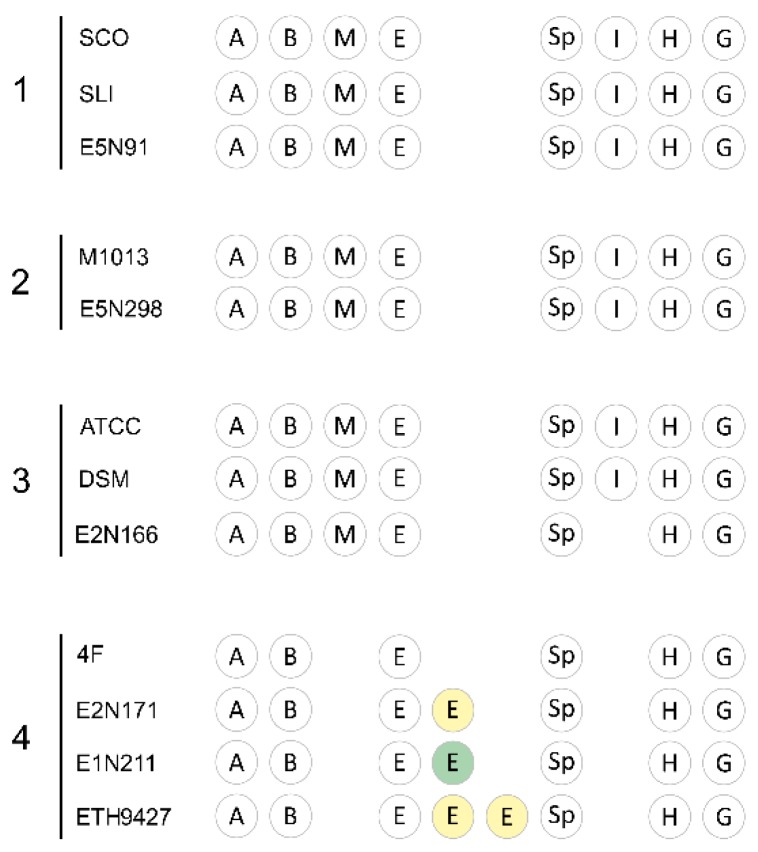
The core specialized metabolites produced by the strains. From the total number of SMBGCs that were identified, the core metabolome constitutes a set of gene clusters frequently conserved across *Streptomyces* species. The letters stand for the specialized metabolites albaflavenone (A), desferrioxamine B (B), melanin (M), ectoine (E), spore pigment (Sp), isorenieratene (I), hopene (H), and geosmin (G). Cluster disposition is not related to chromosomal position. The ectoine clusters in yellow and green represent different gene clusters.

Some of the identified gene clusters are well known and usually found in *Streptomyces* spp. The core SMBGCs are gene clusters that are present in the majority of streptomycetes and in the examined strains were selected based on conservation, with at least 58% of the strains sharing all of the core SMBGCs that encode for: melanin [27], ectoine osmolytes [28], spore pigment [29], the siderophore desferrioxamine B [30], the terpenes albaflavenone [31], hopene [32], isorenieratene [33], and the volatile geosmin [34]. This set of eight biosynthetic gene clusters is present in all of the strains from clades 1, 2, and 3, although no isorenieratene gene cluster was found in strain E2N166 (Figure 3). Interestingly, none of the strains in clade 4 possess either melanin or isorenieratene encoding gene clusters. Furthermore, strains E2N171, E1N211, and ETH9427 have additional ectoine gene clusters: two in ETH9427, and one in each of the other two strains. These have different gene contents (Figure 3, yellow and green E clusters), and as expected, the phylogenetically closer E2N171 and ETH9427 strains have the same extra ectoine encoding gene cluster, that in the case of ETH9427 is doubled due to its location on the chromosomal terminal inverted repeats. This result is consistent with strains from the same species, which usually share the same core metabolome [35].

**Figure 4:**
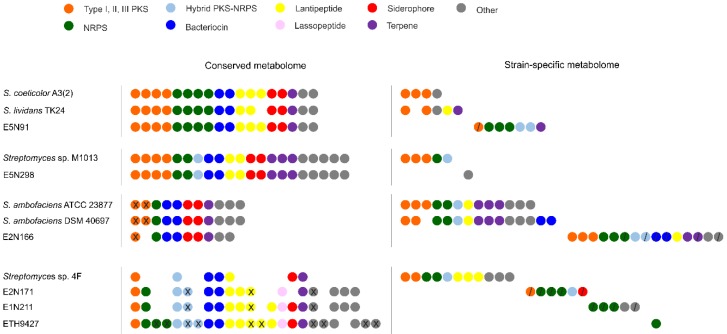
Conserved and strain-specific biosynthetic gene clusters. Within each clade, conserved and strain-specific gene clusters (circles) were identified. Original chromosomal organization is not considered, and clusters with the same position are identical. Colors represent the major classes of specialized metabolites: Type I, II, and III PKS (orange), NRPS (green), hybrid PKS–NRPS (light blue), bacteriocin (dark blue), lantipeptide (yellow), lassopeptide (pink), siderophore (red), terpene (purple), and others that include other KS pathways and less common metabolites such as phenazines, butyrolactones, and indoles (grey). Unknown clusters are indicated with “/” and duplicated clusters with “X”.

In addition to the core metabolome, other SMBGCs are conserved within each clade (Figure 4). Strain E5N91 contains 18 conserved gene clusters out of 33 in total, which are also present in *S. coelicolor* in clade 1. Surprisingly, biosynthetic gene clusters for the characteristic metabolites coelichelin [36], coelibactin [37], calcium-dependent antibiotic [38], and coelimycin [39] are conserved in strain E5N91, whereas the actinorhodin [40] and undecylprodigiosin [41] gene clusters are not, even if they are present in phylogenetically closely related species. In clade 2, two-thirds of the gene clusters identified in strain E5N298 are conserved in *Streptomyces* sp. M1013 (21 out of 30 in total). Conversely, in clade 3, strain E2N166 has only nine gene clusters out of 31 in total conserved with *S. ambofaciens* strains ATCC 23877 and DSM 40697. These include the species-distinctive Type II PKS gene cluster encoding kinamycin [42], the NRPS biosynthetic gene cluster for coelichelin, two siderophore and two bacteriocin gene clusters, and a terpene-encoding gene cluster [43]. The E2N171, E1N211, and ETH9427 strains in clade 4 have a remarkably high number of conserved SMBGCs, with each strain showing 54 to 72% of the identified gene clusters that are present in one or both of the other two strains. However, only seven gene clusters are conserved in *Streptomyces* sp. 4F, which is consistent with its taxonomic relationship in clade 4. These results indicate that phylogenetic relationships are accompanied by specialized gene cluster maintenance.

### 2.4. Strain-Specific Biosynthetic Gene Clusters

Other gene clusters exist in each of the six examined strains beyond the core and conserved metabolome, the strain-specific SMBGCs. These were determined as gene clusters that are exclusive to a given strain and absent from its phylogenetically closest relatives, based on the nucleotide identity of putative biosynthetic genes. The putative products of these strain-specific biosynthetic pathways comprise all the major classes of specialized metabolites (Figure 4). Each strain harbors at least one strain-specific gene cluster. The fewest strain-specific gene clusters were encountered in strains E5N298 and ETH9427, with only one each. Strains E5N91 and E5N298, which are taxonomically close to their clade’s counterparts, dedicate almost opposite amounts of their specialized metabolome to strain-specific products (21% and 3%, respectively), perhaps as a result of horizontal gene transfer events and reflecting different levels of adaptation [44,45]. Conversely, other strains show a significantly higher number of strain-specific gene clusters. Strains E2N171 and E2N166 harbor six and 15 strain-specific gene clusters that represent 21% and 48% of their specialized metabolome, respectively.

**Figure 5:**
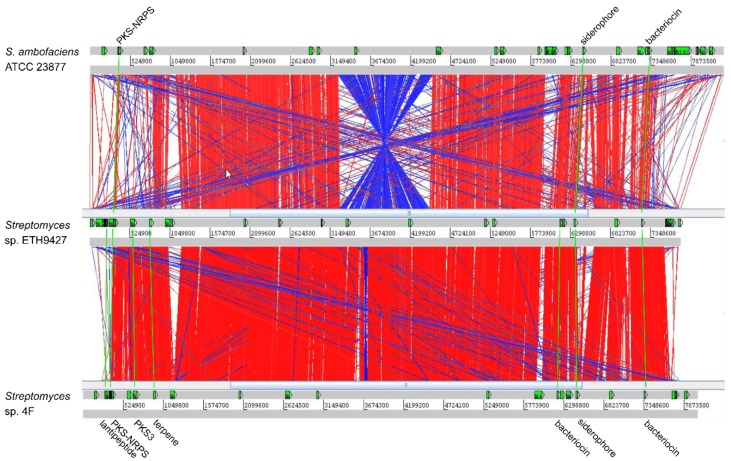
Genome alignment and specialized metabolome comparison of *Streptomyces* sp. ETH9427. Alignment was performed with the Artemis Comparison Tool software (ACT) using an identity threshold of 77% and a score threshold of 500. Synteny regions are represented by red lines, inversion events are represented in blue, and breaks in synteny are seen as blank spaces. SMBGCs are marked in the horizontal panels (green arrows), and conserved gene clusters are linked (green lines).

The preliminary results of genome alignment and sequence comparison of the relatively close E2N166 and *S. ambofaciens* strains reveals large chromosome reorganizations events, such as a pericentral inversion, and most of the acquired strain-specific gene clusters are unexpectedly located in the central region of the chromosome. Moreover, genome alignment and comparison of the ETH9427 strain with its closest related strain *Streptomyces* sp. 4F and the reference species *S. ambofaciens* ATCC 23477 shows a relatively conserved chromosome organization, particularly between the first two strains (Figure 5). The identified SMBGCs also reflect the shared chromosomal regions, with seven gene clusters shared between ETH9427 and *Streptomyces* sp. 4F, most of which are also maintaining relative positions, and only three gene clusters between ETH9427 and *S. ambofaciens* ATCC 23477 (Figure 5), supporting the notion that the distribution of SMBGCs is coherent with phylogenetic relationships [46].

While some of the identified strain-specific gene clusters have orthologues in other species, four out of the six strains that are used in this work (strains E5N91, E2N166, E2N171, and E1N211) have between one and six unknown biosynthetic gene clusters. These represent 3% to 19% of the metabolome, leading to a total of 16 SMBGCs that could possibly lead to novel chemistry. The strain-specific gene clusters identified in E2N171 and E1N211 (six NRPS, a type I PKS and a siderophore-like and another gene cluster classified as “other”) are of particular interest, as they can conceivably be responsible for the antimicrobial activities previously observed in Benhadj et al., namely against nosocomial infections responsible bacterial strains of *Escherichia coli*, *Streptococcus aureus* and *Pseudomonas aeruginosa*. These bioactivities are exclusive to E2N171 and E1N211 amongst the entire collection, and are absent from strains such as E5N298; therefore, the identified strain-specific SMBGCs in these two strains are good candidates for further detailed characterization [13].

## 3. Materials and Methods

### 3.1. Strains and DNA Extraction

Five strains from a previously built *Streptomyces* collection were used in this work [13]. In brief, isolates were obtained from water samples of Lake Fetzara (Annaba, Algeria) on different agar media supplemented with antifungals. Additionally, a strain from the ETH collection (Eidgenosische Technische Hochschule Kultursammlungen, Zurich, Switzerland), *Streptomyces* sp. ETH9427 (accession numbers CP029624, CP029625, CP029626), was also analyzed. Genomic DNA was extracted from liquid cultures using a standard “*salting out*” protocol [47].

### 3.2. Whole-Genome Sequencing and Annotation

Draft genomes were constructed by assembling reads from an Illumina Genome Analyzer (Illumina, San Diego, CA, USA), with pair-ends (c.300 bp) and/or mate-pairs (c.8 kb) DNA fragments libraries, and were processed as described in Thibessard et al. [14]. Sequences were trimmed to eliminate adaptor sequences, and contigs with a size smaller than 1000 pb were eliminated. Draft genomes of the strains E5N91, E2N166, E2N171, E1N211, and E5N298 were submitted into the NCBI GenBank database and given the following accession numbers: RAIE00000000, RAIF00000000, RAIH00000000, RAII00000000, and RAIG00000000, respectively. Whenever possible, sequence assembly and contig ordering were achieved using the CLC Main Workbench (Qiagen, Hilden, Germany), and coding sequence prediction and annotation were automatically performed using the NCBI Prokaryotic Genome Annotation Pipeline (PGAP) [48]. Genome alignments were performed with Artemis Comparison Tool (ACT) [49]. Average nucleotide identity (ANI) was determined using the JSpeciesWS server with the BLAST algorithm (http://jspecies.ribohost.com/jspeciesws/) [19].

### 3.3. Phylogenetic Analysis and Biosynthetic Gene Cluster Identification

The phylogenetic relationships between strains were reconstructed with an MLST analysis [50], using DNA sequences from *atpB*, *gyrB*, *recA*, *rpoB*, and *trpB* genes. Several reference *Streptomyces* species were included in the analysis: *S. ambofaciens* ATCC 23877 (accession number CP012382.1), *S. ambofaciens* DSM 40697 (accession number CP012949.1), *S. coelicolor* A3(2) (accession number NC_003888.3), *S. lividans* TK24 (accession number CP009124.1), *Streptomyces* sp. M1013 (accession number MQUH00000000.1), and *Streptomyces* sp. 4F (accession number CP013142.1). The actinobacteria *Corynebacterium glutamicum* ATCC 13032 (accession number BA000036.3) was used to root the phylogenetic tree. Nucleotide sequence alignments were performed with SeaView software [51] using the muscle algorithm, and a concatenated gene sequence (20592 nt in total) was used to construct a neighbor-joining phylogenetic tree with Kimura’s 2-parameter distance correction and 100 bootstrap replicates with MEGA7 software [52]. Overall, there was a good separation and statistical support for most of the branches in the tree. Biosynthetic gene clusters were predicted using the antiSMASH 3.0 server (https://antismash.secondarymetabolites.org/#!/start) without ClusterFinder algorithm [26].

## 4. Conclusions

The efforts for *Streptomyces* environmental strains genome sequencing continue to show the genus SMBGC’s diversity. Strain de-replication of isolates can be achieved using multi-gene sequence comparison instead of the traditional 16S rRNA, and the analysis of isolated strains reveals a pool for novel specialized metabolites in taxonomically closely related strains. Encountered gene cluster diversity supports phylogenetic relationships, where closely related strains share a higher number of SMBGCs. Horizontal gene transfer as a source of genetic diversity does not distort phylogenetic examination, as these are rare events, and the presence or absence of a SMBGC is not enough to blur phylogenetic relationships. Strain-level analysis has already been demonstrated to lead to the identification of new biosynthetic pathways. Though the strain-specific gene clusters identified here require further characterization, they nonetheless reflect that chemical richness can be found at this level, and imply that the deep sequencing of strains belonging to same species will continue to produce originality.

## Figures and Tables

**Table 1:** Strains analyzed in this work.

Strain	G + C Content (%)	Genome Size (Mb)
E5N91	71.9	8.51
E2N166	70.2	7.91
E2N171	72.3	7.00
E1N211	72.1	7.32
E5N298	71.9	7.87
*Streptomyces* sp. ETH9427	72.1	7.75

**Table 2:** Average nucleotide identity values of the compared strains.

	*SLI*	*E5N91*	*M1013*	*E5N298*	*ATCC*	*DSM*	*E2N166*	*E2N171*	*E1N211*	*ETH9427*	*4F*
*SCO*	99.0	97.3	91.5	91.3	86.9	86.9	87.5	81.7	81.8	81.9	81.8
*SLI*		97.7	91.8	91.6	87.0	87.0	87.7	81.6	81.7	81.8	81.7
*E5N91*			91.2	91.0	86.6	86.6	87.4	81.4	81.4	81.6	81.3
*M1013*				98.2	87.1	87.1	87.9	81.5	81.5	81.7	81.4
*E5N298*					87.2	87.2	87.9	81.7	81.7	81.8	81.6
*ATCC*						99.0	88.9	81.8	81.7	81.8	81.9
*DSM*							88.8	81.7	81.6	81.7	81.9
*E2N166*								82.1	82.1	82.2	82.0
*E2N171*									99.0	99.1	94.6
*E1N211*										99.9	94.4
*ETH9427*											94.1
